# Impact of removal and restriction of me-too medicines in a hospital drug formulary on in- and outpatient drug prescriptions: interrupted time series design with comparison group

**DOI:** 10.1186/s13012-019-0924-0

**Published:** 2019-07-24

**Authors:** Raquel Vázquez-Mourelle, Eduardo Carracedo-Martínez, Adolfo Figueiras

**Affiliations:** 1 Sub-Directorate-General, Galician Health Service (Servicio Gallego de Salud - SERGAS), Galicia Regional Authority, Santiago de Compostela, Galicia Spain; 2 Santiago de Compostela Health Area Authority, Galician Health Service, Santiago de Compostela, Galicia Spain; 3 Department of Preventive Medicine and Public Health, Faculty of Medicine, University of Santiago de Compostela and Consortium for Biomedical Research in Epidemiology and Public Health (CIBER en Epidemiología y Salud Pública - CIBERESP), Santiago de Compostela, Galicia Spain

**Keywords:** Hospital formulary, Low molecular weight heparin, LMWH, Programme efficiency, Pharmacy and therapeutics committee, Prescription drugs, Cardiovascular drugs

## Abstract

**Background:**

The study covered in- and out-of-hospital care in a region in north-western Spain. The intervention evaluated took the form of a change in the hospital drugs formulary. Before the intervention, the formulary contained four of the five low molecular weight heparins (LMWHs) marketed in Spain. The intervention consisted of withdrawing two LMWHs (bemiparin and dalteparin) from the formulary and restricting the use of another (tinzaparin), leaving only enoxaparin as an unrestricted prescription LMWH.

Accordingly, the aim of this study was to evaluate the effect on in- and outpatient drug prescriptions of removing and restricting the use of several LMWHs in a hospital drugs formulary.

**Methods:**

We used a natural, before-after, quasi-experimental design with a control group and monthly data from January 2011 to December 2016. Based on data drawn from official Public Health Service sources, the following dependent variables were extracted: defined daily doses (DDD) per 1000 inhabitants per day (DDD/TID), DDD per 100 stays per day, and expenditure per DDD.

**Results:**

The two compounds that were removed from the formulary registered an immediate decrease at both an intra- and out-of-hospital level (66.6% and 55.6% for bemiparin and 73.0% and 92.2% for dalteparin, respectively); similarly, the compound that was restricted also registered an immediate decrease (36.1% and 9.0% at the in- and outpatient levels, respectively); in contrast, the remaining LMWH (enoxaparin) registered an immediate, significant increase at both levels (44.9% and 32.6%, respectively). The intervention led to an immediate reduction of 6.8% and a change in trend in out-of-hospital cost/DDD; it also avoided an expenditure of €477,317.1 in the 21 months following the intervention.

**Conclusions:**

The results indicate that changes made in a hospital drugs formulary towards more efficient medications may lead to better use of pharmacotherapeutic resources in its health catchment area.

**Electronic supplementary material:**

The online version of this article (10.1186/s13012-019-0924-0) contains supplementary material, which is available to authorized users.

## Introduction

The viability of public health services and their financing is one of the major global health policy debates [1–3], due to the constant increase in costs [4]. Pharmaceutical provision accounts for 25–30% of the public budgeting of healthcare expenditure [5] and is considered to be the main cause of inefficiency in public health services [1].

The main factors involved in high pharmacy costs are the appearance of new drugs, the intensification of treatments, polytherapy, or their prices [6], the promotion of pharmaceutical companies [7, 8], and the induced prescriptions of hospital doctors to primary care doctors [9–11].

Hospital drugs formularies are lists of drugs drawn up to optimise inpatient care and ensure clinically appropriate, safe and cost-effective access [12, 13], and are a common tool for rational drug use in developed countries [14–16]. The growing supply of available drugs, many of which are so-called “me-too drugs” or drugs from the same family, and intense promotion by the pharmaceutical industry has made it necessary to limit these lists [7]. Although drugs formularies are not strictly mandatory, adherence to them by hospital prescribers tends to be very high [15–18], ranging at maximum from 85% to 90% [16].

To our knowledge, there is little research [19–21] evaluating the impact of a change in hospital formularies on outpatient prescription. Hence, the aim of this study was to evaluate the impact of withdrawing two low molecular weight heparins (LMWHs) from a hospital formulary (and restricting a third to the case where the only unrestricted prescription LMWH is contraindicated) on in- and outpatient LMWH prescribing.

## Material and methods

### Scope

The study was carried out in Galicia, a region in north-western Spain which had 2,718,525 inhabitants in 2016. Ninety-nine percent of the population is covered by the Spanish National Health Service (SNHS) with a public health insurance system; a quarter of the population is over 65 years of age. The range of services offered to citizens includes preventive, diagnostic, therapeutic, rehabilitative, and health promotion and maintenance activities.

Visits to the doctor are free of charge; out-of-hospital pharmaceutical service is subject to a financial contribution (co-payment), whereas in-hospital service is free of charge.

In Spain, the official prices of state-funded medicines dispensed in community pharmacies are set by the State. In the study period, the price of LMWHs did not change. In the hospital setting, each hospital can individually negotiate with the pharmaceutical industry the price to be paid for a drug and each hospital freely defines its own hospital drugs formulary for inpatient use.

By virtue of the regulations in force during the study period, at an out-of-hospital level in Spain, regional governments are not empowered to decide which medicines are subject to prior authorisation: this can only be done by the central government.

Spain currently has ~ 11,800 state-funded drugs dispensable under official medical prescription at community pharmacy outlets. Both the funding (degree of reimbursement) and price are set by the central government. There is no published set of rules and regulations governing the criteria applied. Once a medication has been placed on the market, its price tends to remain unchanged until its period of exclusivity expires: it is only then that the price tends to drop due to competition from generic drugs. Spain has a reference price system that categorises drugs according to their active ingredients [22]. There are no formularies in primary care.

Unlike the situation in retail pharmacy outlets, the number of medications included in the hospital drug formulary of these regions’ health service does not usually exceed 1000. These drugs are selected by the Pharmacy and Therapeutics Committee and then directly negotiated between the laboratory and hospital pharmacy department concerned.

In Galicia, around 30% of the health budget for 2017 was allocated to pharmaceuticals as follows: 20% to drugs dispensed at community pharmacies and about 10% to hospital pharmacies.

On the basis of the body of available scientific evidence, the different LMWHs cannot be said to display different degrees of efficacy/effectiveness for their indications, with these drugs being regarded as equivalent treatments [23, 24]. Nevertheless, their out-of-hospital costs per defined daily dose (DDD) are very different. The cost/DDD of each LMWH used to calculate the trend in expenditure per DDD of the set of LMWHs at an ambulatory level was bemiparin, €3.90; tinzaparin, €2.85; enoxaparin, €2.32; nadroparin, €2.24; and dalteparin, €2.12.

### Design

We used a natural, before-after, quasi-experimental design with a control group and monthly data from January 2011 to December 2016. This design not only allows for causal effects to be estimated by controlling for baseline level and trend [25], but it also makes full use of the longitudinal nature of the data, a feature that lends the design its special robustness [26, 27].

The use of a control group in interrupted time series studies (ITS) is not compulsory, since pre- and post-intervention trends within the study population are compared. Even so, a control group can help to reduce confounding caused by external co-interventions. To act as our control group, we therefore selected a health area which had similar characteristics to those of the intervention group and that belonged to the same public health service. This enabled us to control for the possible influence of external factors that affect prescribing, such as the publication of guidelines or articles, alerts pertaining to the medications studied, or pharmaceutical company promotions.

### Intervention group

The Galician Health Service has 7 health areas, one of which is the Santiago de Compostela health area, with a catchment population of 447,699 inhabitants in 2016, a third-level referral hospital with 1043 beds [28], and 74 health centres. Its hospital drugs formulary contained ~ 700 drugs. In the intervention group, a change was made to the LMWHs listed in the hospital drugs formulary in February 2015.

### Control group

The Lugo health area had a catchment population of 308,533 inhabitants in 2016, a third-level referral hospital with 879 beds, and 71 health centres. Its hospital drugs formulary contained ~ 1000 drugs. It was selected as a control group by virtue of having a similar population and healthcare resources [29]. Furthermore, the intervention and control groups belonged to the same public health service.

In the control group, no changes were made during the study period to the LMWHs in the hospital drugs formulary.

### Intervention

The intervention took the form of a change in the hospital drugs formulary. Before the change, the formulary contained 4 of the 5 LMWHs marketed in Spain. The change consisted of removing bemiparin and dalteparin from the drugs formulary of the Clinical University Teaching Hospital in the Santiago de Compostela health area, as well as restricting tinzaparin (i.e., subject to prior authorisation) to very specific cases (creatinine clearance < 30 ml/min). This change took place in February 2015. The only LMWH that remained in the hospital drugs formulary as an unrestricted medication was enoxaparin.

This measure is the consequence of the context described under the head “Scope” regarding out-of-hospital prescription of LMWHs (the cost/DDD of enoxaparin is one of the lowest at retail pharmacy outlets, and its price is non-negotiable in this setting) and the fact that at intra-hospital level, an advantageous reduction was achieved in the unit price of enoxaparin. To our knowledge, there was no other additional intervention implemented by the health authorities in this period.

It should be noted that tinzaparin remained on the formulary because at the time of the change, here in Spain, enoxaparin was contraindicated in patients with severe renal failure. This was not the case with tinzaparin. However, in March 2017, Spain was brought into line with other European countries, with this contraindication being removed from the Spanish summary of enoxaparin product characteristics. Future modifications of the hospital drugs formulary could include the withdrawal of tinzaparin.

### Data sources

The data were drawn from official Public Health Service sources. These sources are of an administrative nature, which implies that the recording of data is population-based (there is no sampling) and exhaustive (there is practically no risk of under-reporting), with the data being linked to accounting and economic aspects of community and hospital pharmacies.

The Official Hospital Pharmacy Information System (known by its Spanish acronym “SINFHOS”) was used for in-hospital data: it shows the purchases made by SNHS hospital pharmacy services for inpatient use.

Data on out-of-hospital prescriptions were obtained from the Official Pharmacy Information System for Complex Pharmacy Service Analyses (known by its Spanish acronym “SIAC_PF”): which shows all drugs dispensed under official medical prescription by community pharmacies in the health area and charged to the SNHS.

### Patient and public involvement

No patients were involved.

### Definition of variables

The number of DDD [30] per month was calculated for each of the five LMWHs marketed in Spain, i.e., bemiparin, dalteparin, enoxaparin, nadroparin, and tinzaparin [31]. A DDD is the assumed average maintenance dose per day for a drug used for its main indication in adults [32].

Accordingly, we calculated the number of DDD per 1000 inhabitants per day (DDD/TID) and the number of DDD per 100 stays per day [33]. These two internationally used basic units of consumption make it possible to examine drug use at both an ambulatory and hospital level in time sequences, without this being affected by changes in such drug’s commercial presentation [33].

For the purpose of calculating DDD/TID, the mean population of the study areas was taken into account, and for the purpose of calculating DDD per 100 stays per day, the mean hospital stays were taken into account. For both DDD/TID and DDD per 100 stays per day, values were calculated for each month over the study period. Expenditure per DDD was calculated by dividing the entire monthly cost by the number of DDD prescribed that month.

### Statistical analysis

For statistical analysis purposes, we used an interrupted time series (ITS) [27, 34] analysis and constructed a segmented linear regression model for each independent variable analysed.

Adjusting for the values of the control area will show the effects of the intervention, by eliminating the possible influence of external co-interventions. To this end, we used the following equation:

$$ {Y}_t={\beta}_0+{\beta}_1{T}_t+{\beta}_2{X}_t+{\beta}_3{\mathrm{X}}_{\mathrm{t}}{\mathrm{T}}_t+{\beta}_4Z+{\beta}_5{\mathrm{ZT}}_t+{\beta}_6{\mathrm{ZX}}_t+{\beta}_7{\mathrm{ZX}}_t{\mathrm{T}}_t+e $$

where

- *Y*_*t*_ is the dependent variable with monthly values (DDD/TID, DDD per 100 stays per day, and expenditure per DDD);
- *β*_0_ represents the initial level of the dependent variable;
- *β*_1_ is the slope of the dependent variable until the implementation of the intervention;
- *T*_*t*_ is the number of months since the start of the study;
- *β*_2_ represents the change in the level of the dependent variable, which occurs in the period immediately following as compared to the period immediately preceding the implementation of the intervention;
- *X*_*t*_ is a dummy variable representing the intervention (pre-intervention period, 1; post-intervention period, 0);
- *β*_3_ represents the difference between the pre- and post-intervention periods in the slope of the dependent variable;
- X_t_T_t_ is an interaction term;
- *β*_4_ represents the difference in the level of the dependent variable between the intervention area and the control area in the pre-intervention period;
- *Z* is a dummy variable that denotes cohort assignment (intervention = 0, control = 1);
- *β*_5_ represents the difference in slope of the dependent variable between the intervention area and the control area in the pre-intervention period;
- ZT_*t*_ is an interaction term;
- *β*_6_ indicates the difference between the control group and the intervention group in terms of the level of the dependent variable immediately after the intervention;
- ZX_*t*_ is an interaction term;
- *β*_7_ represents the difference between the control group and the intervention group in terms of the difference between the slope of the dependent variable after and before the intervention;
- ZX_*t*_T_*t*_ is an interaction term; and,
- *e*_*t*_ is the random error term.

As a measure of adjustment of the statistical model, we used the value of R_*2*_; to control for possible autocorrelation, auto-regressive terms were introduced into the model.

Based on the regression coefficient *β*_6_, we calculated the percentage reduction in the use of LMWHs with respect to the situation immediately preceding the change in the hospital drugs formulary.

To assess the influence of the control group on the results, we performed a sensitivity analysis, by applying a classical ITS model (without a control group).

## Results

Table 1 shows the population distribution of the intervention and control groups.

**Table 1:** Intervention and control group: demographic data

Characteristics	Intervention group	Control group
	*N* = 445,474	*N* = 317,527
Age—*n* (%)		
0–18	67,347 (15.1)	40,825 (12.9)
19–30	47,669 (10.7)	31,163 (9.8)
31–50	134,660 (30.3)	88,068 (27.7)
51–70	119,066 (26.7)	88,665 (27.9)
> 70	76,732 (17.2)	68,806 (21.7)
Sex—n (%)		
Male	215,653 (48.4)	154,065 (48.5)
Female	229,821 (51.5)	163,462 (51.4)

### In-hospital use

Table 2 shows the results of the segmented regression. The monthly trend in DDD/100 stays-day for LMWHs at the hospital where the hospital drugs formulary intervention took place showed that

1. the compounds removed from the formulary [bemiparin and dalteparin] registered an immediate significant decrease: 66.6% for bemiparin (*p* < 0.05) and 73.0% for dalteparin (*p* < 0.01);
2. the compound that was restricted [tinzaparin] also experienced an immediate significant decrease of 36.1% (*p* < 0.01) and a shift from an upward trend before the change to a long-term downward trend (*p* < 0.01); and,
3. the LMWH that remained in the formulary as the first choice [enoxaparin] displayed a sharp change in consumption, going from a previous downward trend to an immediate increase of 44.9% (*p* < 0.01).

**Table 2:** Results of controlled interrupted segmented regression of time series

	Pre-intervention trend		Post-intervention			
			Immediate impact of the formulary change		Change in trend after the formulary change	
	Coefficient	95% confidence interval	Coefficient	95% confidence interval	Coefficient	95% confidence interval
DDD/TID						
Total low molecular weight heparins	− 0.00368	− 0.01652 to 0.00916	− 0.22475	− 0.84762 to 0.39812	0.03792	− 0.00526 to 0.08110
Enoxaparin^a^	− 0.02868*	− 0.03783 to − 0.01953	0.83432*	0.40110 to 1.26754	0.06759*	0.03701 to 0.09817
Bemiparin^b^	0.00739*	0.00165 to 0.01313	− 0.44664*	− 0.70761 to − 0.18567	− 0.02817*	− 0.04681 to − 0.00953
Tinzaparin^c^	0.013880*	0.00651 to 0.02125	− 0.26654*	− 0.45942 to − 0.07366	− 0.00187	− 0.01381 to 0.01007
Dalteparin^b^	− 0.00071*	− 0.00136 to − 0.00006	− 0.02773*	− 0.05092 to − 0.00454	0.00141	− 0.00012 to 0.00294
Nadroparin^d^	0.00062*	0.00005 to 0.00119	0.00459	− 0.01871 to 0.02789	− 0.00043	− 0.00200 to 0.00114
DDD/100 stays and day						
Total low molecular weight heparins	− 0.27284	− 0.91964 to 0.37396	14.51816	− 13.64694 to 42.68326	− 1.80935	− 3.80400 to 0.18530
Enoxaparin^a^	− 0.40854	− 1.01540 to 0.19832	41.83919*	15.89330 to 67.78508	− 0.20613	− 2.10147 to 1.68921
Bemiparin^b^	− 0.24035*	− 0.42506 to − 0.05564	− 8.06595*	− 15.39245 to − 0.73945	− 0.88081*	− 1.42010 to − 0.34152
Tinzaparin^c^	0.51274*	0.33291 to 0.69257	− 12.50797*	− 16.85966 to − 8.15628	− 0.51714*	− 0.82116 to − 0.21312
Dalteparin^b^	−0.06482*	− 0.09016 to − 0.03948	− 1.25262*	− 2.15101 to − 0.35423	0.04139	− 0.01988 to 0.10266
Nadroparin^d^	− 0.00018	− 0.00098 to 0.00062	0.01117	− 0.02974 to 0.05208	0.00016	− 0.00268 to 0.00300
Outpatient expenditure per DDD						
Total low molecular weight heparins	0.00517*	0.00395 to 0.00639	− 0.11977*	− 0.17028 to − 0.06926	− 0.00786*	− 0.01139 to − 0.00433

^a^This was the only one that remained as a non-restricted prescription low molecular weight heparin in the formulary after the intervention

^b^After the intervention, this low molecular weight heparin was removed from the formulary

^c^After the intervention, this low molecular weight heparin was restricted to situations where enoxaparin could not be used

^d^This low molecular weight heparin had already been removed from the formulary before the intervention

*p < 0.05

With respect to the sum of antithrombotic drugs other than LMWHs, there were no significant differences (*p* > 0.05).

### Outpatient use

**Fig. 1:**
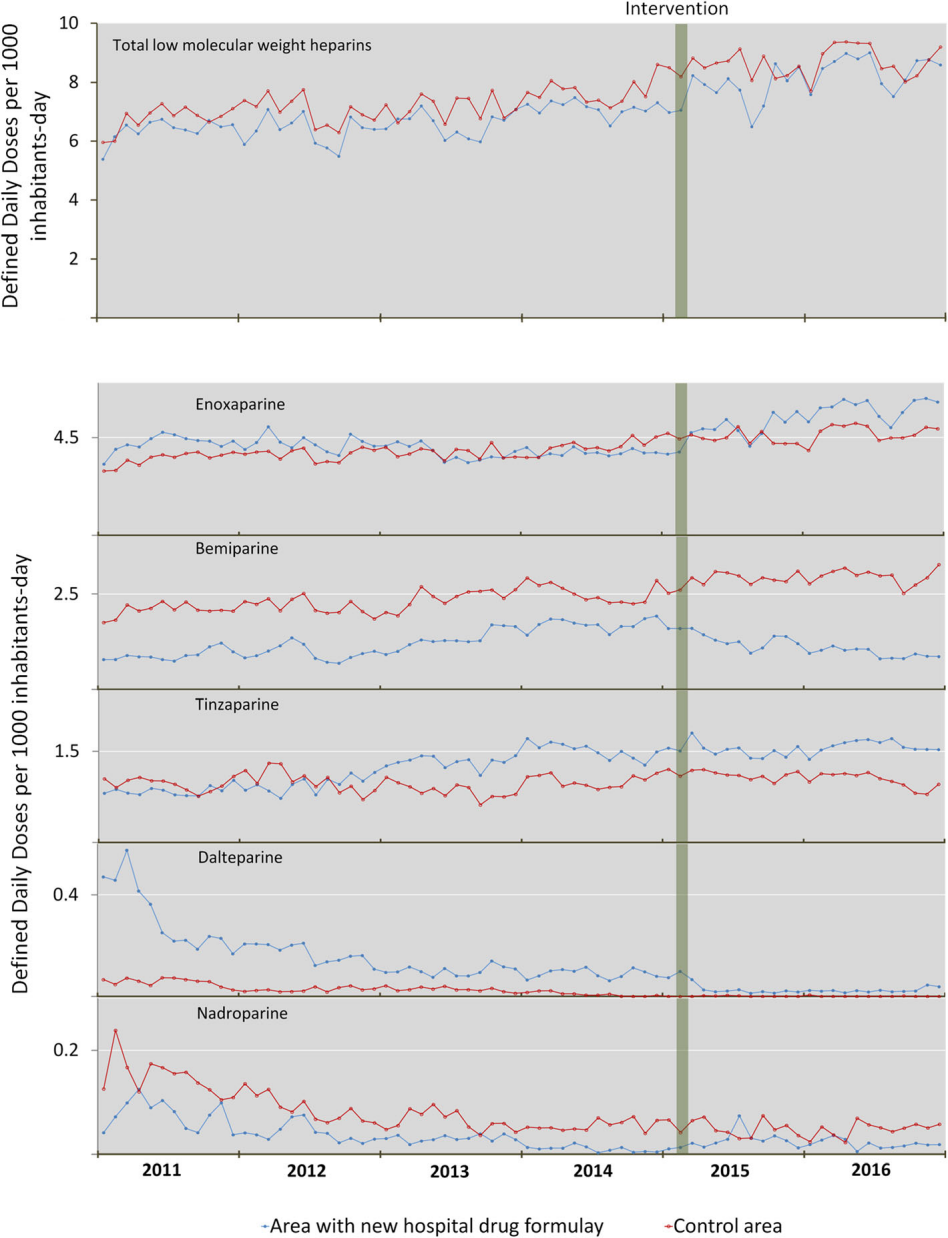
Trends in out-of-hospital low molecular weight heparin use

Figure 1 depicts the monthly trend in out-of-hospital DDD/TIDs for the sum of the five LMWHs and for each individually.

Table 2 shows the results of the segmented regression.

1. In the case of the LMWHs that were withdrawn from the hospital drugs formulary, the effect of the intervention on bemiparin use was to change its previous upward trend (*p* < 0.05) to an immediate significant reduction of 55.6% (*p* < 0.01) and a long-term downward trend (*p* < 0.01). Dalteparin, which had shown decreasing use before the change to the hospital drugs formulary (*p* < 0.05), registered an immediate significant reduction of 92.2% post-intervention (*p* < 0.05).
2. In the case of the LMWH that was restricted (subject to prior authorisation)[tinzaparin], its consumption pattern shifted from a previous upward trend (*p* < 0.01) to an immediate significant reduction of 9.0% post-intervention (*p* < 0.01).
3. Use of the only LMWH remaining in the hospital drugs formulary as an unrestricted prescription drug (enoxaparin) changed sharply from a previous long-term downward trend (*p* < 0.01) to an immediate significant increase of 32.6% (*p* < 0.01) and a long-term upward trend post-intervention (*p* < 0.01).
4. The only LMWH marketed which was not included in the in-hospital formulary before the change was the one with the lowest out-of-hospital use (nadroparin). Indeed, it displayed a downward trend. The modification to the hospital drugs formulary resulted in no statistically significant change in this drug’s use (*p* > 0.05).

Similarly, the sum of all antithrombotic drugs other than LMWHs showed no significant change after the formulary modifications (*p* > 0.05).

### Results in the costs of both scenarios

**Fig. 2:**
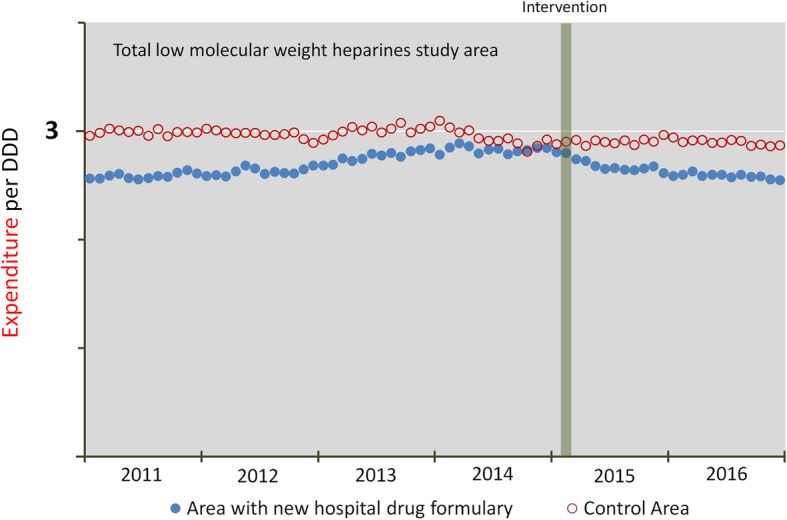
Out-of-hospital expenditure per defined daily dose (DDD)

Most of the total gross cost of LMWHs during the study period (95%) was attributable to out-of-hospital use (with average monthly gross expenditure being €13,454 for in-hospital use versus €256,980 for out-of-hospital use). In contrast to the in-hospital level, at the out-of-hospital level, costs were correlated with drug use. Figure 2 shows the monthly expenditure per DDD of the sum of LMWHs consumed out-of-hospital in both areas. The upward trend in the intervention area in the period preceding the change (*p* < 0.01) was followed by an immediate significant decrease of 6.8%, (*p* < 0.01) and a long-term downward trend after the intervention (*p* < 0.01).

**Fig. 3:**
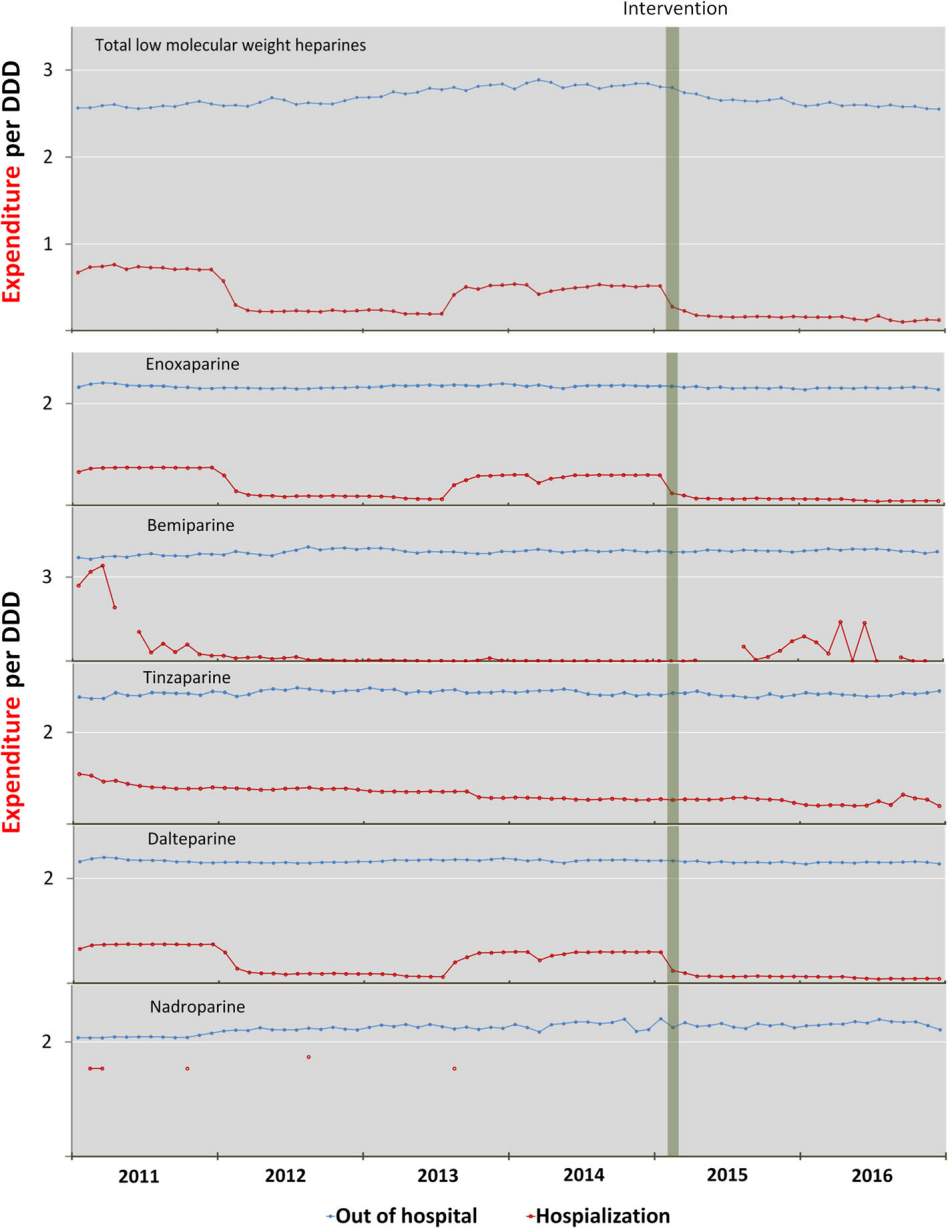
Out-of-hospital and in-hospital expenditure per defined daily dose (DDD) in the intervention area

Figure 3 illustrates expenditure on DDD in- and outpatient levels in the area of hospital intervention; the cost of DDD was much lower at the in-patient than at the outpatient level and displayed large variations throughout the study period, something that did not occur in the outpatient setting. The change in the formulary led to a savings of €477,317.1 for the SNHS over the 21 months analysed post-intervention. The expenditure avoided in this way corresponded almost entirely to the out-of-hospital setting.

### Sensitivity analysis

Additional file 1: Table S1 lists the results of the segmented regression, which shows the effect of the intervention without the control group [26]. The pattern observed was the same, in terms of both direction and significance, as seen in the analysis with the control group.

## Discussion

This is the first longitudinal controlled study to show that in-hospital LMWH prescribing has a major impact on out-of-hospital prescribing.

Our study intervention (which was in-hospital) led to an immediate decrease of almost 8% and a shift from upward to downward in the long-term trend of out-of-hospital expenditure per DDD. These results may well be of great interest to policymakers, as they indicate that hospitals are an important inducer of prescribing in primary care and, by extension, that changes in a hospital drugs formulary towards more efficient drugs can help reduce pharmaceutical costs, not only at an in-hospital but also at an out-of-hospital level [9, 10, 11, 19, 20, 35].

Our data indicate that the withdrawal of drugs from a hospital drugs formulary has an impact on out-of-hospital prescribing, both in the short and in the long term. An example of this is provided by bemiparin: in addition to registering an immediate reduction in use, it showed a shift in trend, from upwards before its removal from the hospital formulary to downwards thereafter. Similarly, the use of dalteparin, which showed a previous downward trend, registered an immediate significant reduction after the drug’s withdrawal.

Although there may be some residual use of some withdrawn medications, this is in line with other studies which indicate that adherence to drugs formularies by hospital health professionals, albeit very high (80–95%) [15–17], is not 100%.

Our data indicate that the introduction of drug-use restrictions in a hospital formulary is associated with a reduction in that drug’s use at both an in- and out-of-hospital level. This is the case of tinzaparin, which, after being made subject to prior authorisation, immediately registered a 45.5% reduction in consumption in the hospital environment. These results are in line with studies that report a sharp decrease in the use of drugs rendered subject to prior authorisation [36–41].

However, in addition to the restriction’s impact on in-hospital prescriptions, there was also an impact outside the hospital, with an immediate 9% reduction in tinzaparin use, despite the fact that the need for prior authorisation was not implemented at the outpatient level. Since, we were unable to locate any other study which analysed the impact of making a drug subject to prior authorisation at the in-hospital level on the prescription of that same drug at the out-of-hospital level (when, at the hospital level, such a drug was not subject to prior authorization), this would make our study the first to analyse such an impact.

At the outpatient level, nadroparin (the only LMWH that was not included in the hospital drugs formulary before the change) experienced no significant changes after the intervention. Moreover, nadroparin is the LMWH with the lowest out-of-hospital use, which further reinforces the theory that in-hospital prescribing has an influence on out-of-hospital prescribing, and is a similar result to that of other cross-sectional studies which report that, when hospitals include a given LMWH in the hospital drugs formulary, there is also greater use of that LMWH at the out-of-hospital level [19].

The finding that 95% of total SNHS expenditure on LMWHs corresponds to out-of-hospital use may be due to the fact that the number of ambulatory patients in the health area is much higher than the number of patients who are hospitalised at any given time and that out-of-hospital use is usually chronic, while in-hospital use tends to be acute. Furthermore, expenditure per DDD at the in-hospital level is lower than at the out-of-hospital level, as it can be subject to discounts. This would explain why the savings yielded by the change to the hospital drugs formulary during the study period correspond almost entirely to the out-of-hospital setting. Biosimilar enoxaparin was not yet being marketed in Spain by the time our study ended; however, once biosimilar enoxaparin [42–44] is marketed in Spain, the savings could be even greater. The fact that costs correlate with consumption at an out-of-hospital but not at an in-hospital level and the large variations in expenditure per DDD in the in-hospital setting (see Fig. 3) may both be related to the discounts offered by pharmaceutical companies to hospitals, as described in other studies [11, 14].

The results of our study indicate that the withdrawal of inefficient drugs from a hospital drugs formulary at an out-of-hospital level and the application of out-of-hospital costs when deciding on the inclusion of a drug in the hospital drugs formulary are aspects that should be taken into account by the committees that decide on the composition of such formularies. Although gross in-hospital spending on a group of drugs may be very small compared to what is spent on an out-of-hospital basis for the same group (e.g., LMWHs, anti-hypertensives or proton pump inhibitors), in-hospital formulary changes may have a considerable induced impact on outpatient drug spending.

Pharmacy and Therapeutics Committees play a major role in establishing a local, safe, and effective drug policy [45–47], yet we found no studies addressing how and when a drug should be withdrawn from a hospital drugs formulary. Our results suggest incorporating the withdrawal of inefficient drugs as another essential function. In this connection, it should be noted that this function of evaluating the withdrawal of drugs from the hospital drugs formulary comes at practically zero cost to the health organisation.

To our knowledge, this is the only controlled longitudinal study to analyse the effect of removing a compound from a hospital drugs formulary in two care scenarios, i.e., inpatient and outpatient, and shows that the effect of hospital-induced prescribing on the primary care setting can be modified to guide the latter towards a more efficient drug provision.

The fact that this study was controlled had the advantage that, if there had been changes in consumption or external factors (publications of guides, articles, commercial pressure, safety alerts), this would have been similar in both health areas. In addition, ITS analyses have the advantage that confounding is seldom a problem, since population characteristics (structure by age, sex, educational level, and disease prevalence) tend to change gradually over an extended period of time [48, 49].

In some cases, the intervention and control groups are not perfectly parallel in the pre-intervention period. Here, the data of other potential control groups did not serve as a comparator for our study, in that they did not have the drug which was withdrawn and caused the change in the formulary. To assess the influence of the control group on the results, we conducted a sensitivity analysis by applying a classical ITS model (without a control group). The results were found to be very similar, something that goes to reinforce this study’s conclusions.

As in any observational study with drugs, the absolute value of the variables DDD per day-stay and cost per DDD is limited by the characteristics of hospitals, in terms of activity and care provision. However, we believe that the effects on the trend over time in their use can be extrapolated.

## Conclusion

Given that the resources of health systems are limited and that pharmaceutical expenditure is a high percentage of their expenditure, hospital drug formularies can be a very useful and effective tool for improving out-of-hospital prescription profiles in an area and can contain the cost increases associated with drug provision. This is especially important when one recalls that the evaluation of a given drug’s removal comes at zero cost to the organisation. This measure can be implemented in any setting that has a hospital drugs formulary. The hospital can therefore become a generator of good prescribing practices and induce a more efficient use of pharmaco-therapeutic resources in its health catchment area.

## Additional file

Additional file 1: **Table S1.** Results of the interrupted segmented regression without a control group (DOC 52 kb)

## Data Availability

The datasets used and/or analysed during the current study are available from the corresponding author on reasonable request.

## Abbreviations

DDD: Defined daily doses
DDD/100 s-d: Defined daily doses per 100 stays and a day
DDD/TID: Defined daily doses per 1000 inhabitants per day
LMWHs: Low molecular weight heparins
SNHS: Spanish National Health Service
