# Supplementary material for: Impact of removal and restriction of me-too medicines in a hospital drug formulary on in- and outpatient drug prescriptions: interrupted time series design with comparison group
Source: Implement Sci. 2019 Jul 24;14:75. doi: 10.1186/s13012-019-0924-0 (PMC6657080; doi:10.1186/s13012-019-0924-0)
Supplement: Supplementary file 1 — Table S1. Results of the interrupted segmented regression without a control group (DOC 52 kb) [file 13012_2019_924_MOESM1_ESM.doc]

|  |  | | **Table S1** Results of the interrupted segmented regression without a control group | | | | | | | |  |  |
| --- | --- | --- | --- | --- | --- | --- | --- | --- | --- | --- | --- | --- |
|  |  | |  | Pre-intervention  Trend | | Post-intervention | | | | | | |
|  |  |  |  |  |  | Immediate impact of the formulary change | | Change in trend after the formulary change | | | | |
|  |  | | | Coefficient | 95% Confidence Interval | Coefficient | 95% Confidence Interval | Coefficient | | 95% Confidence Interval | | |
|  | **DDD/TID** | | | | | | | | | | | |
|  | Total Low Molecular Weight Heparins | | | 0.01885* | 0.00113 to 0.03657 | 0.61299* | 0.02938 to 1.19660 | -0.01368 | | -0.05568 to 0.02832 | | |
|  | Enoxaparin^a^ | | | -0.01136 | -0.01826 to -0.00446 | 0.84455* | 0.48863 to 1.20047 | 0.05022* | | 0.02239 to 0.07805 | | |
|  | Bemiparin^b^ | | | 0.02217 | 0.01880 to 0.02554 | -0.40494* | -0.57577 to -0.23411 | -0.05006* | | -0.06141 to -0.03871 | | |
|  | Tinzaparin^c^ | | | 0.01508 | 0.00608 to 0.02408 | -0.12652 | -0.25643 to 0.00339 | -0.01144* | | -0.02151 to -0.00137 | | |
|  | Dalteparin^b^ | | | -0.00129* | -0.00207 to -0.00051 | -0.02212 | -0.04405 to -0.00019 | 0.00188* | | 0.00037 to 0.00339 | | |
|  | Nadroparin^d^ | | | -0.00163* | -0.00194 to -0.00132 | 0.03373* | 0.01789 to 0.04957 | 0.00110* | | 0.00004 to 0.00216 | | |
|  | **DDD/ 100 stays and day** | | | | | | | | | | | |
|  | Total Low Molecular Weight Heparins | | | -0.382543 | -0.84650 to 0,08142 | 25.637324* | 2.58738 to 48.68727 | -0.921489 | | -2.48907 to 0.64609 | | |
|  | Enoxaparin^a^ | | | -1.09928* | -1.65966 to -0.53890 | 58.27636* | 35.16116 to 81.39156 | -0.04264 | | -1.50474 to 1.41946 | | |
|  | Bemiparin^b^ | | | 0.14296* | 0.02783 to 0.25809 | -9.07515* | -12.91610 to -5.23420 | -0.16919 | | -0.55466 to 0.21628 | | |
|  | Tinzaparin^c^ | | | 0.23465* | 0.03536 to 0.43394 | -7.12158* | -10.91530 to -3.32786 | -0.18507 | | -0.51874 to 0.14860 | | |
|  | Dalteparin^b^ | | | -0.07769* | -0.09713 to -0.05825 | -1.20878* | -2.20534 to -0.21222 | 0.06667 | | -0.00277 to 0.13611 | | |
|  | Nadroparin^d^ | | | -0.00004 | -0.00022 to 0.00014 | 0.00044 | -0.00667 to 0.00755 | 0.00004 | | -0.00045 to 0.00053 | | |
|  | **Outpatient expenditure per DDD** | | | | | | | | | | | |
|  | Total Low Molecular Weight Heparins | | | 0.00268 | -0.14060 to -0.05800 | -0.09930* | -0.00846 to -0.00086 | -0.00466* | | -0.00846 to -0.00086 | | |
|  | a) This was the only one that remained as an unrestricted prescription low molecular weight heparin in the formulary after the intervention.  b) After the intervention, this low molecular weight heparin was removed from the formulary.  c) After the intervention, this low molecular weight heparin was restricted to situations where enoxaparin could not be used.  d) This low molecular weight heparin had already been removed from the formulary before the intervention.  * *p* < 0.05. | | | | | | | | | | | |
|  | |  | | | | | | |  |  |  |  |
